# Case reports: novel *TUBG1* mutations with milder neurodevelopmental presentations

**DOI:** 10.1186/s12881-019-0827-6

**Published:** 2019-05-31

**Authors:** Yue T. K. Yuen, Ilaria Guella, Elke Roland, Michael Sargent, Cyrus Boelman

**Affiliations:** 1 Faculty of Medicine, University of British Columbia, Vancouver, Canada; 2 Centre for Applied Neurogenetics, University of British Columbia, Vancouver, Canada; 3 Division of Neurology, BC Children’s Hospital, Vancouver, Canada; 4 Department of Medical Imaging, BC Children’s Hospital, Vancouver, Canada

**Keywords:** Microcephaly, *TUBG1*, Tubulin, Malformations of cortical development, Intellectual disability, Epilepsy

## Abstract

**Background:**

Tubulinopathies result from mutations in tubulin genes, including *TUBG1*, responsible for cell microtubules, are characterized by brain development abnormalities, microcephaly, early-onset epilepsy, and motor impairment. Only eleven patients with *TUBG1* mutations have been previously described in literature to our knowledge. Here we present two new patients with novel de novo *TUBG1* mutations and review other cases in the literature.

**Case presentations:**

Both patients have microcephaly and intellectual disability. Patient B further fits a more typical presentation, with well-controlled epilepsy and mild hypertonia, whereas Patient A’s presentation is much milder without these other features.

**Conclusion:**

This report expands the spectrum of *TUBG1* mutation manifestations, suggesting the possibility of less severe phenotypes for patients and families, and influencing genetic counselling strategies.

## Background

Mutations in the tubulin genes (e.g. *TUBA1A, TUBB2A, TUBA8, TUBB2B, TUBB3, TUBB5, TUBG1*) are associated with a range of brain malformations. Common tubulinopathic presentations include an array of lissencephalies, polymicrogyria-like cortical dysplasia, simplified gyral pattern, microlissencephaly, and a dysmorphic corpus callosum [1, 2]. Eleven patients with a *TUBG1* mutation have been described in the literature to our knowledge, and only limited clinical information is available for these patients. They are described with microcephaly, motor impairment, intellectual disability and epilepsy [1, 3, 4]. Here we report two further patients with novel *TUBG1* mutations and milder presentations.

## Case presentations

These two patients are followed at BC Children’s Hospital, a public academic tertiary pediatric referral centre, serving a population of nearly 5 million people.

**Fig. 1:**
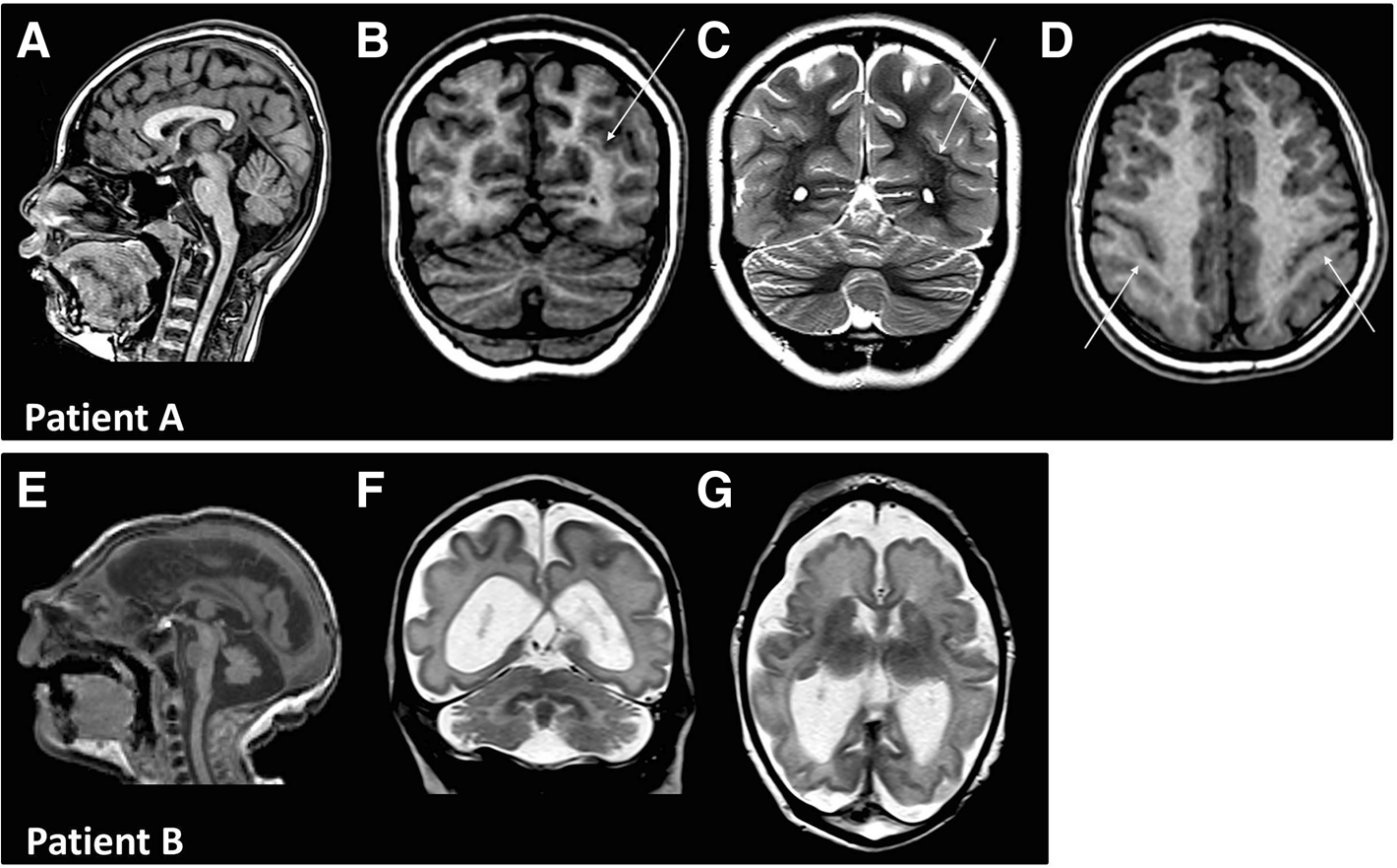
MRI Findings of Patient A with *TUBG1* p.Asp68Asn mutation and Patient B with *TUBG1* p.Arg341Trp mutation. **a** Sagittal T1-weighted MPRAGE image demonstrates reduced craniofacial ratio in this child with microcephaly and posteriorly predominant simplified cortical gyri. The cerebellar vermis is normal. The corpus callosum appears slightly thick. **b** and **c** Coronal (**b**) T1 weighted MPRAGE and (**c**) Coronal T2 weighted fast spin echo images demonstrating bilateral parietal band heterotopia, more pronounced on the left (arrow). **d** Axial T1 weighted MPRAGE image shows bilateral blurring of the grey-white interface along the central sulci, in keeping with focal cortical dysplasia (arrows). **e** Sagittal T1-weighted image demonstrates microcephaly, intact corpus callosum and normal pituitary. The pons and cerebellar vermis are small for gestational age, but are notably less affected than the supratentorial brain. **f** and **g** Coronal (**f**) and axial (**g**) T2-weighted images demonstrating dilated lateral ventricles and simplified gyral pattern. In (**f**) there is prominence of the cerebellar folia but normal-appearing dentate nuclei. In (**g**) the basal ganglia and thalami are small and poorly defined, with absent myelination of the posterior limb of internal capsule

Patient A is a 10-year old right-handed female born following a pregnancy complicated by antenatal microcephaly noted on fetal ultrasound. She is of Chinese descent and has no family history of consanguinity or congenital anomalies. Her early development milestones were normal; she sat at 6 months, crawled at 8 months and walked at 12 months of age. Her mild intellectual disability was first apparent at the age of six years and she is now two years behind her peers academically, with no regression in development. She speaks two languages. She has an independent education plan. At birth her head circumference was not measured and by ten years of age the patient’s head circumference was 46 cm (2 SD below the mean). She has some dysmorphic facial features including prominent ears relative to her microcephaly, a tented mouth, and bilateral 5th finger clinodactyly. Neurological examination was otherwise unremarkable. Magnetic resonance imaging (MRI) at nine years of age revealed microcephaly, posteriorly predominant simplified cortical gyri, and areas of band and nodular heterotopia (Fig. 1). No seizure-like activity has been described by the parents but a screening electroencephalogram (EEG) demonstrated occasional interictal sharp waves over the right central temporal areas in drowsiness and sleep, suggestive of a predisposition towards focal onset seizures, in addition to occasional, non-specific, generalized paroxysmal delta activity in sleep. Chromosomal microarray and biochemical screening for inborn errors of metabolism (as described elsewhere) were both unremarkable [5]. Clinical whole exome sequencing (Centogene AG, Rostock, Germany) revealed a novel, de novo *TUBG1* missense mutation (NM_001070.4: c.202G > A; p.Asp68Asn), using a trio approach (proband plus parents), chosen due to the multiple potential causative genes. The parents consented to this report.

Patient B is a 13-month old male born following a pregnancy complicated by fetal ultrasound findings of microcephaly (bitemporal narrowing), possible cavum septum pellucidum, enlarged ventricles, agenesis of the corpus callosum, and lissencephaly. He is of Caucasian descent with no family history of consanguinity or congenital anomalies, but a maternal history of simple febrile seizures and paternal history of asymptomatic Huntington’s disease gene carriers. Head circumference at birth was below the 3rd percentile, (measured as 21 cm at 2 weeks of age), length 10-50th percentile, and weight 10th percentile. Up-slanting palpebral fissures were noted. Within the first hour of life, he experienced a seizure characterized by left-sided clonic activity with secondary bilateral synchrony and oxygen desaturation. This was managed with phenobarbital. The interictal EEG at this point showed diffuse suppression and excessive left central sharp waves, suggestive of cerebral dysfunction with some focality. A full septic workup was completed and negative. MRI at the age of thirteen days demonstrated microcephaly, a small cerebellum, reduced number and complexity of the cerebral sulci and gyri with no cortical thickening or polymicrogyria, general paucity of cerebral white matter, dilated lateral ventricles, a normal third ventricle, small lentiform nuclei which were not well separated from the small thalami, and a grossly normal corpus callosum for age. There was normal myelination in the brainstem and cerebellum but there was lack of myelination in the posterior limbs of internal capsules. The patient had twenty-nine more seizures within the first forty-three days of life. These were twenty to forty seconds in length and generally associated with feeding. They involved a combination of apnea and cyanosis, lip smacking, head turning to the right, left eye deviation, arm flexion, leg extension, and post-ictal fatigue. At last follow-up, seizures were controlled with levetiracetam and topiramate. He has central hypothyroidism on thyroxine but otherwise normal pituitary function. He was also found to have small optic nerves, and a small secundum atrial septal defect shunting left to right. At 13 months, last follow up, he is not yet rolling or sitting independently, but has head control, brings his hands to the midline, is babbling and visually fixing and following. Neurological examination is notable for slightly low axial tone and increased appendicular tone, with a head circumference of 36 cm (below 3rd percentile). Chromosomal microarray and biochemical screening for inborn errors of metabolism were both unremarkable [5]. Clinical whole exome sequencing (GeneDx, Gaitherburg, USA) revealed a novel, de novo *TUBG1* missense mutation (NM_001070.4: c1021C > T; p.Arg341Trp) using a trio approach. The parents consented to this report.

## Discussion and conclusions

Tubulinopathies are characterized by a wide range of brain malformations, including lissencephaly, polymicrogyria and mildly simplified gyral patterning. The full phenotypic spectrum of tubulinopathies is however not yet fully known. All but one of the eleven reviewed cases of *TUBG1* mutations involved epilepsy, majority of which were refractory and early-onset in nature. Of the seven patients with birth head circumference measurements, six had microcephaly (< 2 standard deviations (SD) below the mean) and four of the six had severe microcephaly (< 3 SD below the mean). Motor dysfunction was present in all but one patient out of the nine with the available data, ranging from delayed motor development to spastic tetraplegia. All patients suffered from intellectual disability ranging from moderate to severe [1, 3, 4].

Patient A represents the least severe manifestation of *TUBG1* mutation reported so far, having microcephaly, brain malformations, mild facial dysmorphia, and mild intellectual disability, but no motor impairment or epilepsy. Patient B had a more typical presentation sharing features with the more severe phenotype: microcephaly, brain malformations, global development delay and early-onset epilepsy (though easily controlled). Other neurological, clinical, and genetic phenotypes are compared in summary in Table 1.

**Table 1:** Clinical, imaging, and genetic features of patients with *TUBG1* mutations

ID	Patient A	Patient B	Patient 1^1,3^	Patient 2^1,3^	Patient 3^1,3^	Patient 4^4^	Patient 5^4^	Patient 6^4^	Patient 7^4^	Patient 8^4^	Patient 9^4^	Patient 10^4^	Patient 11^4^
Mutation	c.202G > A; p.Asp68Asn	c1021C > T; p.Arg341Trp	c.1160 T > C; p.Leu387Pro	c.275A > G; p.Tyr92Cys	c.991A > C; p.Thr331Pro	c.63C > A; p.Phe21Leu	c.985G > T; p.Asp329Tyr	c.776C > T; p.Ser259Leu	c.776C > T; p.Ser259Leu	c.776C > T; p.Ser259Leu	c.776C > T; p.Ser259Leu	c.769A > T; p.Ile257Phe	c.776C > T; p.Ser259Leu
Mode of inheritance	De novo	De novo	De novo	De novo	Father’s DNA N/A	De novo	Father’s DNA N/A	De novo	De novo	Germline mosaicism in parent	Germline mosaicism in parent	De novo	De novo
Mutation effect	Aspartate to asparagine within a highly conserved residue in the GTP-binding pocket	Arginine to tryptophan within a highly conserved residue located in the C-terminal domain that is required for the dimerization of y-tubulin	Leucine to proline within a highly conserved residue within an α-helix located in the C-terminal domain	Tyrosine to cysteine within a highly conserved residue in the vicinity of the GTPase domain	Threonine to proline within a highly conserved residue in an α-helix within the γ-γ protein interaction domain located in the C-terminal domain	Phenylalanine to leucine within the GTPase domain	Aspartate to tyrosine within a highly conserved residue in the C-terminal domain. Located on the surface of the *TUBG1* protein	Serine to leucine within a highly conserved residue located in the C-terminal domain	Serine to leucine within a highly conserved residue located in the C-terminal domain	Serine to leucine within a highly conserved residue located in the C-terminal domain	Serine to leucine within a highly conserved residue located in the C-terminal domain	Isoleucine to phenylalanine within a highly conserved residue located in the C-terminal domain	Serine to leucine within a highly conserved residue located in the C-terminal domain
Sex	F	M	F	M	F	M	M	F	F	F	M	M	F
Age at follow-up	10y	6mo	21y	18mo	31y	33y	21y	19mo	14y	11y 6mo	9y 6mo	15y	18mo
Head circumference	<−2.1 SD	<−1.9 SD	<−5.5 SD	<−4 SD	<−1 SD	57 cm	53.1 cm (<−2.6 SD)	<−3.5 SD	N/A	47.5 cm at 6y 6mo (<−3.3 SD)	N/A	51.3 cm at 13y (<− 2.5 SD)	N/A
Epileptic	No	Yes	Yes	Yes	Yes	Yes	Yes	No	Yes	Yes	Yes	Yes	Yes
Seizure age of onset	–	< 1 h of life	Early-onset	N/A	Early-onset	36mo	N/A	–	6mo	4mo	N/A	3y 11mo	5mo
Type of seizures	–	Focal with secondary bilateral synchrony	N/A	Infantile spasms	N/A	Tonic-atonic-myoclonic	Partial complex; versive, myoclonic	–	Tonic-clonic	Generalized tonic-clonic	N/A	N/A	Focal, versive
Refractory epilepsy	–	No	Yes	Yes	Yes	N/A	Yes	–	N/A	N/A	N/A	N/A	No
Motor dysfunction	No	Mild axial hypotonia; appendicular hypertonia	Spastic tetraplegia (bedridden)	Spastic tetraplegia (bedridden)	Moderate cerebral palsy	Spastic tetraplegia (walks with support)	Spastic tetraplegia	Delayed motor development	Unsteady gait	Spastic diplegia	N/A	N/A	Delayed motor development
ID	Moderate	Moderate global delay	Severe	Severe	Moderate	Severe	Severe	N/A	N/A	Moderate	Moderate	Moderate (FS IQ-score 44)	Severe
Speech and Language Development	Normal	Moderate global delay	N/A	N/A	N/A	Only sounds, no speech	Non-verbal	Delayed	Non-verbal	50 words	Non-verbal	5–6 word sentences	Non-verbal
Age at MRI	9y	13 days	N/A	N/A	N/A	36y	11y	1y 6mo	12mo	13y 7mo	2mo	6y	9y
Cortical dysgenesis (MRI)	Posterior predominant pachygyria, band heterotopia, nodular heterotopia	Reduced cortical sulci and gyri	Severe posterior predominant pachygyria/agyria (posterior agyria, frontal pachygyria), thick cortex	Severe posterior predominant pachygyria/agyria (posterior agyria, frontal pachygyria), thick cortex	Posterior pachygyria, moderate posterior subcortical band heterotopia	Posterior predominant pachygyria (posterior frontal lobe and parieto-occipital cortex)	Diffuse agyria	Posterior predominant pachygyria (mild over frontal lobes, moderate over posterior lobes), cortex 10-13 mm thick	Posterior predominant pachygyria, sparse cells over occipital lobes, cortex 13-15 mm thick	Posterior predominant pachygyria (mild over frontal lobe, moderate over temporal and occipital lobes), cortex 6-13 mm thick	Posterior predominant pachygyria (mild over frontal lobe, moderate over temporal and occipital lobes), cortex > 15 mm thick	Posterior predominant pachygyria (almost normal over frontal lobes, pachygyria over perisylvian and occipital lobes), cortex 6-10 mm thick	Posterior predominant pachygyria (mild over frontal lobe, moderate over temporal and occipital lobe, deep parietal lobe infolding)
Corpus callosum (MRI)	Normal-Thick	Normal	Thin	Thick, dysmorphic	Thick, dysmorphic	Normal	Thin	Normal	Normal	Normal	Thin	Normal	Thin
Other MRI Findings	–	General paucity of white matter, small cerebellum, dilated lateral ventricles, small lentiform nuclei, small thalami, posterior limbs of internal capsule lacking myelination	Mildly enlarged lateral ventricles, mildly reduced white matter	Mildly enlarged lateral ventricles, severely reduced white matter	–	Enlarged perivascular spaces, enlarged posterior horns of lateral ventricles, hippocampal malrotation	Severely enlarged lateral ventricles, severely reduced white matter, dysplastic basal ganglia, hypoplastic brainstem, hypoplastic vermis	Mildly enlarged lateral ventricles, mildly reduced white matter	Mildly enlarged lateral ventricles, mildly reduced white matter	Enlarged posterior horns of lateral ventricles	Mildly enlarged lateral ventricles	Mildly enlarged posterior horns of lateral ventricles, mildly reduced white matter	Mildly enlarged lateral ventricles, mildly reduced white matter, dysplastic basal ganglia

*F* female, *M* male, *y* years, *mo* months, *SD* standard deviation, *ID* intellectual disability, − absent, *N/A* not available

Whereas tubulin gene mutations often follow an autosomal dominant mode of inheritance [2], *TUBG1* mutations are almost entirely *de novo* [1, 3, 4]. Furthermore, tubulinopathies encompass a range of phenotypes including extreme lissencephaly, severe cerebellar hypoplasia, and varying cortical thickness [6, 7]. Despite overlap between the brain phenotypes of different tubulinopathies, certain phenotypes are associated with specific tubulin genes [8]. *TUBG1* patients, are characterized by pachygyria/agyria that is most intense in the parieto-occipital regions with a posterior to anterior gradient as well as enlarged lateral ventricles and reduced white matter volume [3, 4]. Lastly, unlike other tubulinopathies, majority of *TUBG1* mutation patients have a normal cerebellum, basal ganglia, and brainstem [1, 4]. Patient A coincides with the known *TUBG1* presentation involving posteriorly-predominant pachygyria and a normal cerebellum, basal ganglia, and brainstem. However, this patient diverges from the known *TUBG1* mutation phenotype due to the presence of band and nodular heterotopia, normal white matter volume, and normal lateral ventricle size. Patient B is similar to known *TUBG1* mutation in having pachygyria (although not posteriorly-predominant), dilated lateral ventricles, reduced white matter, normal cerebellum, and brainstem. Unlike typical *TUBG1* mutation phenotype, this patient has a small cerebellum. In consideration of the microcephaly spectrum, radiologically, Patent B may correspond to a label of congenital microcephaly with a simplified gyral pattern [9].

*TUBG1* encodes for γ-tubulin, highly expressed in the developing fetal brain as a component of centrosomes. It plays an integral role in microtubule nucleation, thereby affecting microtubule-dependent mitosis and brain development [3, 4]. Poirier et al. introduced mutations in the γ-tubulin gene (*tub4*) in *S. cerevisiae* which interfered with microtubule nucleation*.* Suppression of *TUBG1* in utero of mice also arrested neuronal migration [3]. Depending on the mutation locus, the different *TUBG1* mutations are thought to have an effect on γ-tubulin structure or function. It is suspected that *TUBG1* mutations affect neuronal migration hence MRI findings showing pachygyria/agyria. This is in comparison to other tubulinopathies which are more associated with polymicrogyria or dysgyria [4].

**Fig. 2:**
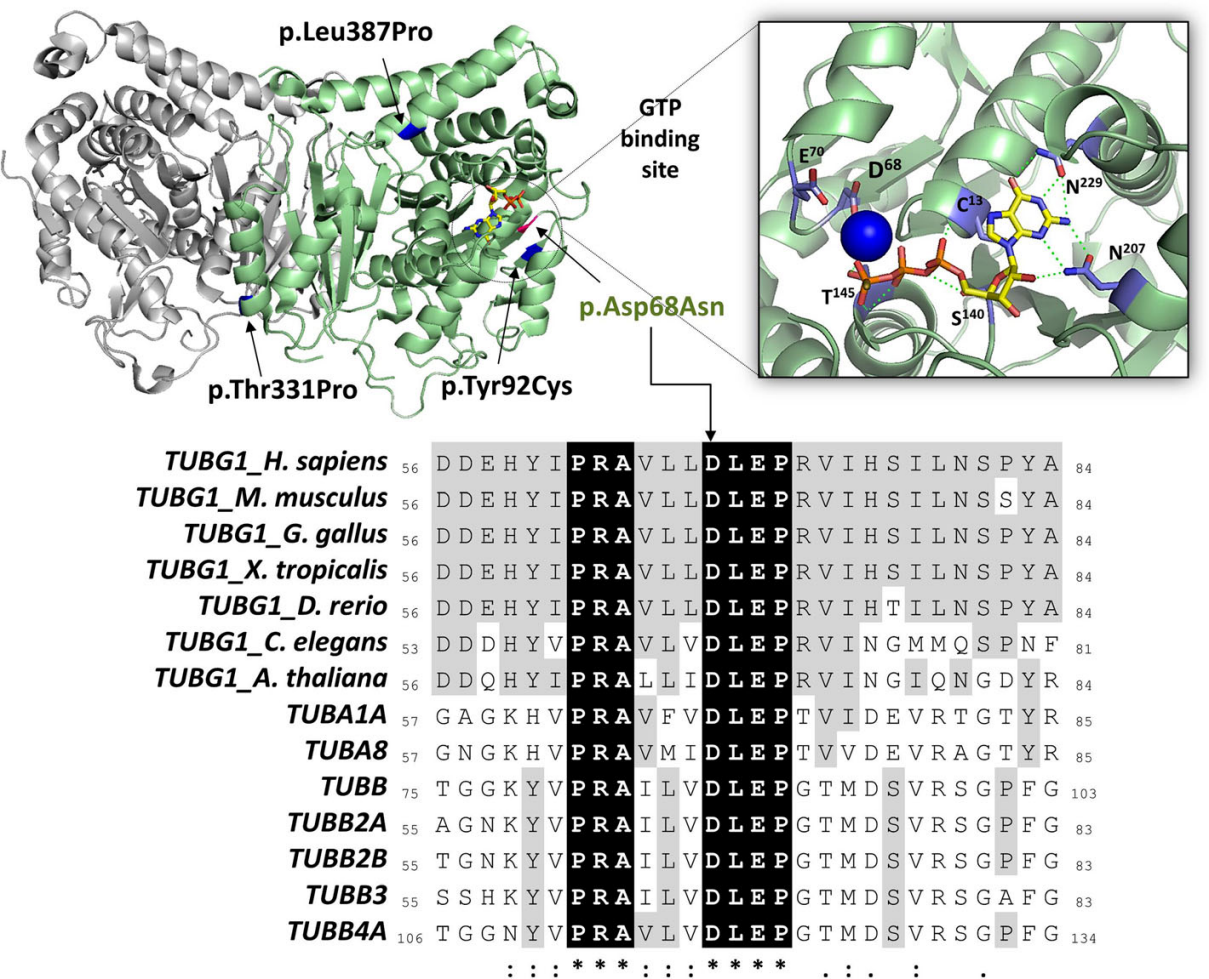
*TUBG1* Mutations in Individuals with Refractory Early-Onset Epilepsy. Top: crystal structure of γ-tubulin. Dimeric γ-tubulin is shown as ribbons, and the GTP bound molecule is shown as stick (PDB ID: 3CB2 [13]). Mutated residues, identified in this study (green) are shown. Right: close-up view of the GTP-binding pocket. Left: close-up view of the γ-γ dimer interface. GTP molecule and interacting residues are shown in stick representation, the Mg^2+^ ion as a sphere, and hydrogen bonds as green dashed lines (PDB ID: 1Z5V [7]). Images were generated using PyMOL. Bottom: partial sequence alignment of *TUBG1* orthologs and different human tubulin proteins surrounding the Asp68 mutated residue. Identical residues across all proteins are shown in black, and residues identical to the human *TUBG1* are in gray. GenBank accession numbers are as follows: *Homo sapiens*, NP_001061.2; *Mus musculus*, NP_598785.1; *Gallus gallus*, XP_015155127.1; Xenopus tropicalis, NP_001072509.1; Dario rerio, NP_957202.1; *Caenorhabditis elegans*, NP_499131.1; *Arabidopsis thaliana*, NP_191724.1; human *TUBA1A*, NP_001257328.1; human *TUBA8*, NP_061816.1; human *TUBB*, NP_001280141.1; human *TUBB2A*, NP_001060.1; human *TUBB2B*, NP_821080.1; *TUBB3*, NP_006077.2; and human *TUBB4A*, NP_001276052.1. Sequences were aligned with CLUSTAL Omega.32 Asterisks indicate positions with a single fully conserved residue, colons indicate conservation between groups with strongly similar properties, and periods indicate conservation between groups with weakly similar properties

We suspect the *TUBG1* mutations found to be likely pathogenic. Both missense mutations described in this paper were *de-novo* resulting in substitutions in highly conserved amino acids which are predicted to be damaging by multiple *in-silico* algorithms, including SIFT, PolyPhen-2, MutationTaster2. Neither mutation has been reported in public databases (gnomAD Browser), and two likely pathogenic entries are present in ClinVar for the Arg341 residue (Patient B). The p.Asp68Asn mutation (Patient A) affects a highly conserved residue in all 20 human tubulin proteins and their ancestral homologs. The Asp68 residue is located in the GTP-binding pocket and together with the Glu72 coordinates the Mg2+ ion that interacts with the GTP γ-phosphate [10, 11] (Fig. 2). The p.Arg341Trp mutation (Patient B) substitutes a positively-charged arginine with an aromatic amino-acid, within the Tubulin/FtsZ 2-layer sandwich (or C-terminal) domain (Fig. 2). The Arg341 residue is part of a stretch of polar amino acids (Arg339, Arg341, Glu342, Arg343 and Lys344) at the interface of γ–γ tubulin dimer and plays a critical role in the in assembly of the γ tubulin homodimer by forming multiple H-bonds with residue Asp252 on the opposite chain [12].

We present here two patients each with a novel, de novo *TUBG1* mutation with common features of microcephaly and intellectual disability but lacking both spastic tetraplegia and more severe refractory epilepsy. The especially mild phenotype of patient A expands the spectrum associated with *TUBG1* mutations and will provide a much different perspective in genetic counselling to families moving forward. However, further work is needed to more fully understand genotype-phenotype correlations in this rare genetic disorder.

## Abbreviations

EEG: Electroencephalogram
MRI: Magnetic resonance imaging
SD: Standard deviation
